# From insult to hyperexcitability: pharmacological targeting of MyD88 and JAK/STAT3 pathways in epilepsy

**DOI:** 10.1007/s10787-026-02210-9

**Published:** 2026-04-13

**Authors:** Ahmed M. Abdelaziz, Mohamed N. Fawzy, Mohamed K. Fathy, Mustafa M. Shokr

**Affiliations:** 1 Department of Pharmacology and Toxicology, Faculty of Pharmacy, Sinai University–Arish Branch, Arish, 45511 Egypt; 2 Department of Pharmaceutics, Faculty of Pharmacy, Sinai University–Arish Branch, Arish, 45511 Egypt

**Keywords:** Neuroinflammation, Mitochondrial dysfunction, Epileptogenesis, Metabolic therapies

## Abstract

Neuroinflammation is hypothesized to be a fundamental driver of epileptogenesis, potentially contributing to the transformation of the healthy brain into a state prone to spontaneous recurrent seizures. This manuscript explores the pivotal roles of the pro-inflammatory cytokines interleukin-1β (IL-1β) and interleukin-6 (IL-6) in modulating neuronal excitability and structural plasticity. We delineate how the activation of the NLRP3 inflammasome and the P2×7 receptor pathway leads to the maturation of IL-1β, which subsequently triggers the MyD88 and PI3K/AKT/mTOR cascades. These pathways collectively enhance NMDA receptor activity and glutamate release while suppressing GABAergic inhibition, establishing a cycle of neuronal hyperexcitability. Furthermore, we examine the systemic and local impacts of IL-6 signaling mediated through the JAK/STAT3 pathway. Beyond acute synaptic effects, IL-6 contributes to chronic pathology by inducing gliosis, hindering hippocampal neurogenesis, and promoting blood-brain barrier leakage via CCL2 production. These multi-level disruptions not only facilitate seizure activity but also contribute to the cognitive and behavioral comorbidities often observed in epilepsy. By synthesizing current understanding of these signaling axes, this review highlights the therapeutic potential of targeting specific cytokine receptors, such as the IL-1 receptor antagonist, to intercept the epileptogenic process. Understanding these neuroinflammatory benchmarks is essential for developing disease-modifying treatments that move beyond symptomatic seizure control toward true prevention of epilepsy.

## Introduction

Epileptogenesis is generally viewed as an active, multistep process initiated through some sort of initial insult to the brain, followed by a latent silent period, and evolving to eventual chronic epilepsy characterized by spontaneous seizures (Park 2024). During this evolution, acute molecular changes in ion channels, receptors, and gene expression are gradually transformed into persistent alterations in synaptic connectivity, network organization, and glial function. Mechanistically, the development of epilepsy is characterized by an imbalance between excitation and inhibition promoted by factors such as the loss of inhibitory interneurons, aberrant axonal sprouting, or dysfunctional astrocyte or microglial signaling (Park 2024; Badawi et al. 2024a). The latter is further systematized with continuously expressed inflammatory signaling and metabolic stress, lowering the seizure threshold and predisposing to propagating, self-reinforcing systems hyperexcitability (Park 2024).

Mitochondrial function is crucial for seizure control, given that neurons heavily depend on oxidative phosphorylation to maintain ionic gradients and support synaptic activities (Xie et al. 2024). Defective mitochondrial metabolism is associated with ATP depletion, which may facilitate hypersynchronous discharges characteristic of seizures. In primary mitochondrial diseases, as well as in other metabolic disorders, epilepsy is a frequent manifestation that underscores a tight link between energy failure and epileptogenesis (Xie et al. 2024; Shokr 2025a). Beyond the issues of inherited disease, acquired mitochondrial stress during status epilepticus or following brain injury can generate reactive oxygen species (ROS), disrupt calcium homeostasis, and trigger cell death pathways that further destabilize neural circuits (Xie et al. 2024).

Neuroinflammation is now considered a key driver of epileptogenesis, which is mediated by the activation of microglia, astrocytes, and endothelial cells, releasing cytokines such as interleukin‑1 beta (IL-1β), tumor necrosis factor alpha (TNF-α), and high mobility group box 1 (HMGB1) (Shi et al. 2025; Alshahrani et al. 2025a). These mediators modulate synaptic transmission, alter receptor function, and compromise blood-brain barrier (BBB) integrity, collectively increasing neuronal excitability and synchrony. Animal and human studies have shown that inflammatory signaling mechanisms are upregulated within hours to days after a seizure-provoking insult and may persist into the chronic phase, correlating with seizure severity and drug resistance (Łukawski and Czuczwar 2023; Shi et al. 2025; Liu et al. 2025b). Targeting specific inflammatory pathways has reduced seizure frequency and improved outcomes in experimental models, supporting the fact that neuroinflammation is not only a consequence but also a modifiable contributor to epileptogenesis (Table 1) (Łukawski and Czuczwar 2023; Shi et al. 2025).

While numerous mediators, including TNF-α and HMGB1, are established drivers of the early neuroinflammatory response in epilepsy, this manuscript prioritizes the IL-1β/MyD88 and IL-6/JAK-STAT3 axes as central targets. This prioritization is based on their unique roles as mechanistic bridges: IL-1β acts as a primary ‘metabolic-electrical’ switch that directly enhances NMDA receptor activity and activates the PI3K/AKT/mTOR cascade, while IL-6 signaling through the JAK/STAT3 pathway serves as a critical mediator of the chronic structural and functional remodeling, such as gliosis and BBB leakage, that sustains the epileptogenic state. Consequently, these pathways represent the most proximal therapeutic checkpoints for intercepting the transition from acute insult to chronic hyperexcitability (Huang et al. 2025).

During epileptogenesis, mitochondrial dysfunction and neuroinflammation form a self-reinforcing pathogenic loop through bidirectional interactions (Mani et al. 2025). Mitochondria-derived ROS and damage-associated molecular patterns activate innate immune receptors and transcription factors, such as nuclear factor kappa B (NF-κB), promoting the production of pro-inflammatory cytokines. On the other hand, inflammatory mediators impair mitochondrial respiration, increase nitric oxide and oxidative stress, and disrupt metabolic signaling pathways, including AMP-activated protein kinase (AMPK) and mammalian target of rapamycin (mTOR), further exacerbating energy failure and synaptic instability (Mani et al. 2025; Alshahrani et al. 2025b). This crosstalk helps explain why metabolic interventions-for instance, ketogenic diets or mitochondria-protective agents- along with anti-inflammatory strategies show promise as disease-modifying approaches in epilepsy, rather than simply symptomatic antiseizure treatments (Fan et al. 2019).

**Table 1:** A table demonstrates linking key inflammatory mediators in TLE

Cytokine	Evidence of elevation in TLE	Associated clinical outcome	Mechanistic/clinical implication	References
IL-1β	Increase in the epileptogenic hippocampus and CSF	Drug resistance, increased seizure frequency	Enhances neuronal excitability via NMDA receptor phosphorylation and BBB disruption	(Suleymanova and Karan 2025)
IL-6	Elevated in serum and brain tissue of TLE patients	Seizure severity, poor surgical outcome	Promotes gliosis, alters synaptic plasticity, and sustains chronic inflammation	(Giovannini and Meletti 2023)
HMGB1	Translocated and released from neurons and glia	Recurrent seizures, pharmacoresistance	Activates TLR4/RAGE signaling, amplifying neuroinflammation and excitotoxicity	(Xu et al. 2025)

## Mitochondrial dysfunction in epilepsy

### Oxidative stress and energy imbalance

Oxidative stress and energy imbalance represent interrelated mitochondrial dysfunctions that can convert a previously stable neural network into a seizure-prone and epileptogenic one (Kong et al. 2024). In the brain, mitochondria produce ATP mainly by oxidative phosphorylation; this process inevitably generates ROS as by-products; when ROS production overwhelms endogenous antioxidant capacity, oxidative damage to lipids, proteins, and nucleic acids accumulates. Excessive ROS may modify ion channels, receptors, and transporters, alter redox-sensitive signaling pathways, and disrupt calcium homeostasis-all these changes increase neuronal excitability and vulnerability to synchronous firing (Kong et al. 2024; Abulaban et al. 2025). Simultaneously, mitochondrial dysfunction decreases ATP availability, thereby compromising Na+/K+‑ATPase and other ATP-dependent pumps that maintain membrane potential and ionic gradients, thus lowering seizure threshold (Zong et al. 2024; Khowdiary et al. 2025).

This combination of oxidative damage and bioenergetic failure affects not only neurons, but also astrocytes and microglia, thus limiting astrocytic uptake of glutamate and potassium and promoting a pro-inflammatory microglial phenotype, further amplifying network instability (Zong et al. 2024; Alqahtani et al. 2025). Recurrent seizures contribute to worsening the problem acutely because of the surging of metabolic demand and ROS generation, creating in this way a vicious cycle where oxidative stress and energy imbalance act both as cause and effect of the ongoing epileptic activity (Al-Beltagi et al. 2025).

NADPH oxidases (NOXs) emerge as highly regulated, spatially and temporally dynamic contributors to pathological redox signaling, distinct from mitochondrial ROS leakage (Saadi et al. 2022). In particular, NOX2 has been consistently implicated as a central driver of seizure susceptibility and disease progression (Saadi et al. 2022; Singh et al. 2025b). Chemoconvulsant models, including kainic acid and pilocarpine–induced seizures, demonstrate rapid NOX2 activation in neurons and microglia during the ictal and early post-ictal phases, leading to superoxide generation, lipid peroxidation, and redox-dependent activation of NF-κB and NLRP3 inflammasome pathways (Singh et al. 2025b). Pharmacological or genetic suppression of NOX2 attenuates acute seizure severity, dampens microglial cytokine release, and limits mitochondrial dysfunction, highlighting bidirectional amplification between NOX-derived ROS and mitochondrial oxidative stress (Singh et al. 2025a).

Importantly, post–status epilepticus interventions targeting NOX2 reduce neuronal loss and epileptogenesis, with recent evidence indicating sex-dependent effects, where NOX2 inhibition confers greater neuroprotection in males, potentially reflecting hormonal modulation of redox and inflammatory signaling (Singh et al. 2025a). In chronic epilepsy models, sustained NOX2 upregulation in microglia and perivascular cells correlates with ongoing neuroinflammation, BBB disruption, and lowered seizure threshold, underscoring its role beyond seizure initiation (Singh et al. 2022). Collectively, these findings position NOX2 as a nodal enzymatic source of ROS that links cell-type–specific redox signaling to neuroinflammation, mitochondrial injury, and long-term epileptic network remodeling, thereby reinforcing oxidative stress as an active driver rather than a byproduct of epilepsy pathophysiology (Saadi et al. 2022).

### Mitochondrial dynamics and quality control

#### Primary mitochondrial epilepsies

Primary mitochondrial epilepsies arise from inherited defects in nuclear or mitochondrial DNA that directly impair oxidative phosphorylation and mitochondrial homeostasis (Table 2) (Lopriore et al. 2022). Pathogenic variants in genes encoding respiratory chain subunits, mitochondrial tRNA synthetases, or proteins regulating mitochondrial dynamics and quality control (e.g., POLG, TWNK, MT-ATP6) lead to chronic bioenergetic failure in neurons and glia. Because the brain has exceptionally high ATP demand and limited glycolytic reserve, even modest reductions in mitochondrial function can destabilize membrane potentials, impair Na⁺/K⁺-ATPase activity, and alter Ca^2+^ buffering, collectively lowering seizure thresholds (Lopriore et al. 2022). These epilepsies often present early in life, are frequently pharmacoresistant, and are accompanied by multisystem features such as myopathy, lactic acidosis, or neurodevelopmental delay. Mechanistically, seizures are a primary consequence of intrinsic mitochondrial dysfunction, with excessive ROS production, defective mitophagy, and impaired neurotransmitter recycling contributing to network hyperexcitability (Yang et al. 2023). Therapeutically, strategies emphasize metabolic support and mitochondrial rescue, cofactor supplementation, ketogenic or anaplerotic diets, antioxidants, and emerging gene- or enzyme-replacement approaches, rather than conventional antiseizure drugs alone (Lopriore et al. 2022).

#### Secondary mitochondrial stress in acquired epilepsy models

In contrast, secondary mitochondrial stress in acquired epilepsy reflects downstream mitochondrial impairment triggered by external insults such as status epilepticus, traumatic brain injury, stroke, infection, or chronic neuroinflammation (Table 2) (Waldbaum and Patel 2010). In these contexts, mitochondria are initially intact but become dysfunctional due to excitotoxic Ca²⁺ overload, excessive glutamate signaling, oxidative and nitrosative stress, and inflammatory cytokine exposure (Waldbaum and Patel 2010). Recurrent seizures further exacerbate mitochondrial depolarization, disrupt fission–fusion balance, and damage mtDNA, creating a vicious cycle in which mitochondrial failure amplifies neuronal hyperexcitability and epileptogenesis (Waldbaum and Patel 2010). Unlike primary mitochondrial epilepsies, these changes are often region-specific (e.g., hippocampus) and temporally dynamic, evolving from an acute injury phase into chronic epilepsy (Neumann and Britsch 2024). Approaches such as antioxidants, Nrf2 activators, mitophagy enhancers, anti-inflammatory agents, or timely metabolic modulation aim to preserve mitochondrial integrity and interrupt epileptogenic cascades, complementing standard antiseizure therapies rather than replacing them (Neumann and Britsch 2024).

While acquired mitochondrial stress is common in many epilepsies, it must be distinguished from primary mitochondrial epilepsies caused by mutations in genes like *POLG*. In these syndromes, the primary energy failure leads to a distinct clinical phenotype characterized by metabolic crises and stroke-like episodes, requiring metabolic cocktails rather than standard anti-seizure protocols (Lopriore et al. 2022).

**Table 2:** Distinctions between primary and secondary mitochondrial dysfunction in epilepsy

Feature	Primary mitochondrial epilepsy	Secondary mitochondrial stress
Feature	Genetic mutations (mtDNA or nuclear)	Brain insult (SE, TBI, stroke)
Feature	Symptom of primary metabolic failure	Driver of further mitochondrial decay
Feature	Chronic ATP deficit & impaired repolarization	ROS-mediated damage & mt-DAMP release
Therapy focus	Metabolic bypass, gene therapy	Mito-protection, anti-inflammatories

### Links between mitochondrial stress and neuronal hyperexcitability

The links between mitochondrial stress and neuronal hyperexcitability arise from several converging biophysical and signaling mechanisms that ultimately favor synchronous, repetitive firing (Sun and Li 2025). Energy failure reduces ATP-dependent restoration of ionic gradients after action potentials, prolonging depolarization and facilitating repetitive discharges, especially in neurons with high firing rates and extensive axonal arbors (Money et al. 2014). Impaired mitochondrial calcium uptake in presynaptic terminals leads to elevated cytosolic calcium and prolonged neurotransmitter release, increasing glutamatergic drive and network excitation, while also activating calcium-dependent enzymes that can damage membranes and cytoskeletal elements (Duarte et al. 2023; Shokr et al. 2025b). ROS and reactive nitrogen species modify voltage-gated sodium and potassium channels, gamma-aminobutyric acid (GABA_A_) and N-methyl D-aspartate (NMDA) receptors, and gap junctions, shifting the balance toward excitation and synchrony; in interneurons, oxidative and metabolic injury can selectively impair inhibitory circuits, removing a critical brake on principal cell firing (Orfali et al. 2024; Dash et al. 2025).

In addition, mitochondrial stress activates redox and energy-sensing pathways such as AMPK, mTOR, and NF-κB, altering gene expression programs that control synaptic plasticity, receptor composition, and inflammatory mediator production (Liu et al. 2025a; Shokr 2025b). Over time, these acute changes consolidate into structural rearrangements, including aberrant mossy fiber sprouting, altered spine density, and gliosis, which further embed hyperexcitability into the network architecture (Dash et al. 2025; Alshahrani et al. 2025a). Thus, mitochondrial stress is not merely a passive marker of seizure activity but a mechanistic driver that couples metabolic and redox disturbances to enduring changes in neuronal excitability and circuit organization (Liu et al. 2025a).

Mito-inflammatory convergence describes the bidirectional crosstalk between mitochondrial dysfunction and innate immune activation that drives chronic inflammation in aging and disease (Zhang et al. 2025). Damaged mitochondria generate excess ROS, release mitochondrial DNA, cardiolipin, and other danger-associated molecular patterns that activate pattern-recognition receptors, particularly the NLRP3 inflammasome (Zhang et al. 2025). In turn, inflammatory cytokines and immune signaling further impair mitochondrial biogenesis, dynamics, and bioenergetics, creating a self-amplifying pathological loop. This convergence underlies inflammaging, neurodegeneration, metabolic disorders, and cardiovascular disease, highlighting mitochondria-centered anti-inflammatory strategies as promising therapeutic targets (Zhang et al. 2025).

## Neuroinflammatory pathways in epileptogenesis

### Key cellular players: microglia, astrocytes, and endothelial cells

Microglia are the resident innate immune cells of the central nervous system and are known as highly motile sentinels that continuously survey the brain parenchyma, rapidly responding to even subtle changes in neuronal activity (Camberos-Barraza et al. 2024). In the process of epileptogenesis, microglia are activated with the alteration of microscopic appearance and functions, transferring from the surveillant state of the ramified microglia to the amoeboid, phagocytic ones that can be broadly separated into the spectrum ranging from the pro-inflammatory “M1-like” state to the anti-inflammatory “M2-like”, which was simplified (Victor and Tsirka 2020; Aljarba et al. 2025). In the activated state, microglia produce a variety of cytokines and chemokines such as IL-1β, TNF α, interleukin-6 (IL-6), CCL2, and complement factors, and the substances such as the reactive oxygen and nitrogen species with the properties to lower the seizure threshold and the potential to provide the favorable environment for the generation of recurrent seizures (Zeng et al. 2023).

A particular importance presents the production of IL-1β and TNF α, which enhance the release of glutamate, the reduction of the glutamate uptake into the neuron, and the process of the GABA_B_ receptor endocytosis, with the resulting excess of the excitatory state and the synchronization of the neuron networks (Zeng et al. 2023; Shokr et al. 2025a). In microglia, the final stages of the P2 × 7 receptors activated by ATP are expressed, and prolonged activation of the ATP-P2 × 7 pathway during the process of status epilepticus establishes the final stages of microgliosis, activation of the inflammasome NLRP3, and the final production of the cytokines, with the resulting enhancing cycle of the inflammatory process (Tewari et al. 2024). Besides the production of the soluble factors, microglia physically interact with the synapses, pruning the excitatory and the inhibitory synapses with activity dependence (Tewari et al. 2024). In the process of chronic activation, the activity can be deleterious, establishing the final pathophysiological process with the generation of the aberrant connectivity of the epileptic circuit (Paolicelli et al. 2022). Microglia have the potential impact not only on the synapse but also on the process of the dentate gyrus formation during the seizure, with the reduction of the aberrant granule cells formation via the toll-like receptor 9 (TLR9)-dependent TNF-α signaling (Matsuda et al. 2015; Rajab et al. 2025). In addition, the process of the microglia with the activity of the astrocytes and the endothelial cells has been established, with the induction of the “A1” type of astrocytes with the production of substances with triggering properties (Han et al. 2025).

**Fig. 1:**
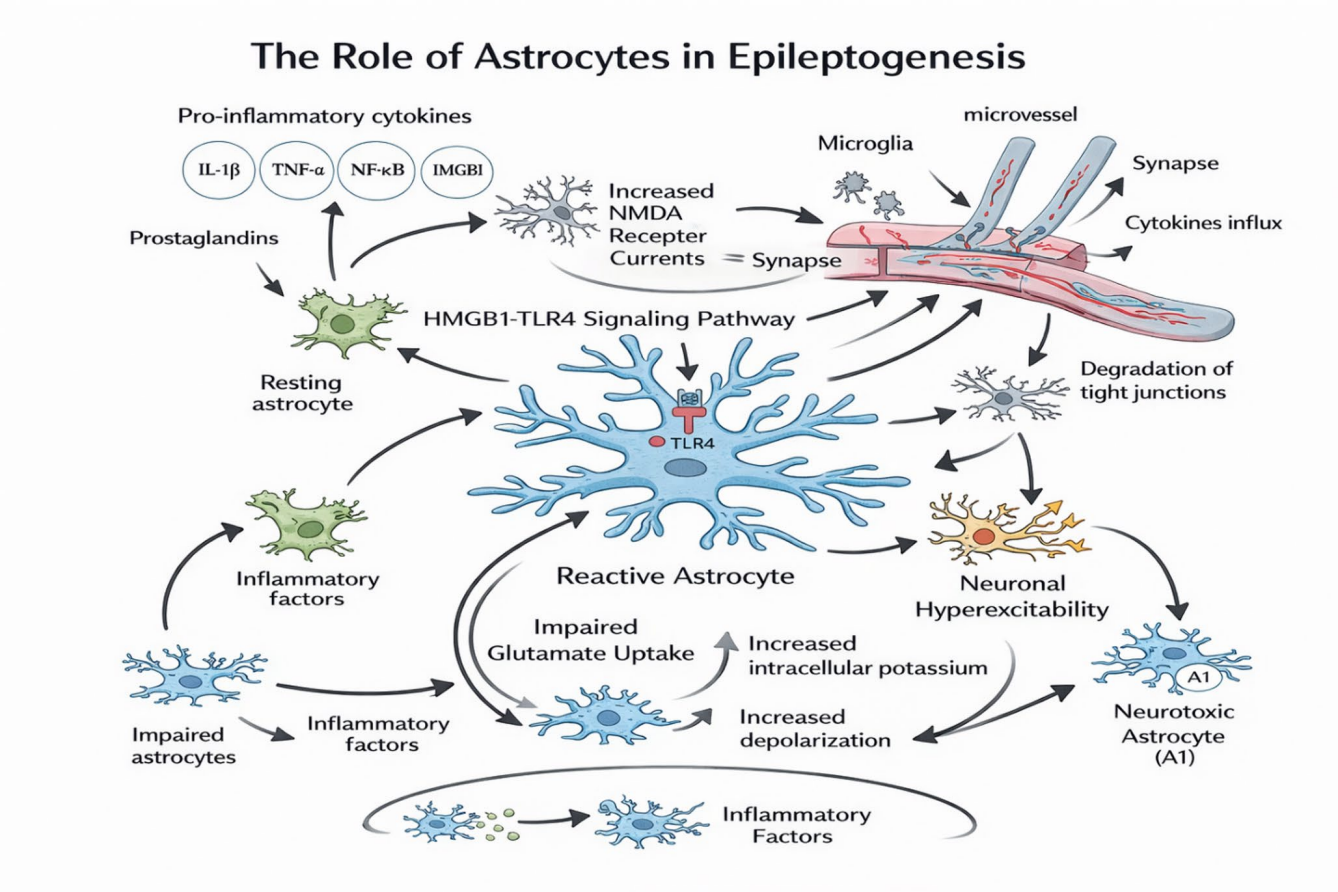
The mito-inflammatory convergence in epileptogenesis. This loop shows how dysfunctional mitochondria and brain inflammation fuel one another to turn a one-time brain injury into a lifelong seizure disorder. The crosstalk between these two forces, driven by key signaling pathways like NF-κB and mTOR, leads to leaky brain barriers and hyperactive neurons. Addressing this trinity of issues with specialized diets and targeted medication offers a way to treat the underlying cause of the disease rather than just the symptoms

The astrocyte represents the largest population of glia within the central nervous system (CNS) and undertakes vital roles in ion homeostasis via buffering, neurotransmitter regulation via reuptake, energy metabolism, and upkeep of the blood-brain barrier (BBB), all of which are strongly perturbed within the context of epileptogenesis (Fig. 1) (Tiwari et al. 2025). Under normal circumstances, the end-feet of astrocytes envelop synapses and microvessels and are subsequently responsible for the uptake of extracellular potassium via inward rectifier potassium channels and the removal of glutamate from the synapse via high-affinity transporters such as GLAST/GLT-1 (Tiwari et al. 2025). The reactive astrocyte (astrogliosis), which takes shape in response to seizures and CNS damage, represents an astrocytic hypertrophy with increased expression of glial fibrillary acidic protein (GFAP), besides undergoing transcriptional regulation into either neuroprotective ‘A2-like’ or ‘A1-like’ astrocytes dependent on the inflammatory context (Giovannoni and Quintana 2020). These reactive astrocytes produce pro-inflammatory cytokines (IL-1β, TNF-α), chemokines, prostaglandins, and HMGB1 proteins that further activates microglia and brings about the influx of leukocytes into the tissue and directly alters neuronal receptors leading to neuronal hyperexcitability; the HMGB1-TLR4 signaling pathway, with particular emphasis on epilepsy with reduced responsiveness to drugs, increases NMDA receptor currents and Ca2 + entry (Giovannoni and Quintana 2020).

Simultaneously, the uptake of glutamate via astrocytic glutamate transporters could be impaired, besides the increased opening of hemichannel and pannexins leading to increased concentrations of extracellular glutamate and ATP levels, thereby driving hyperexcitability and additional microglia activation (Mohamed et al. 2019). The impaired buffering of extracellular potassium associated with altered expression or activity of astrocytic Kir4.1 ion channels leads to excess accumulation of extracellular potassium that depolarizes neurons and thus promotes paroxysmal activity in hippocampal and cortical regions of the brain with seizures (Zhang and Liu 2025). The astrocyte also regulates local energy metabolism through neuronal lactate uptake and neuronal activity regulation with direct coupling to microvessel blood flow; however, these astrocytic functions are impaired under inflammatory stimulation with seizures that result in neuronal energy failure (Beard et al. 2022). The astrocytic end-feet enveloping microvessels also regulate the integrity of the BBB; the reactive astrocyte would thus produce factors that augment BBB integrity besides producing matrix metalloproteinases and vascular endothelial growth factor (VEGF) proteins under chronic inflammatory stimulation, leading to degradation of the tight junctions and increased permeability of the microvessels (Yue and Hoi 2023). The astrocyte thus represents an important focal point where disruptions in ion homeostasis regulation, neurotransmitter regulation, energy metabolism regulation, and inflammatory signaling interact in precipitating the progression from seizures to chronic hyperexcitable neuronal circuits (Yue and Hoi 2023).

The cerebral microvascular endothelium constitutes the main functional unit of the BBB, with a pivotal role in the inflammatory/excitatory milieu of the epileptic brain (Huang et al. 2025). In physiological conditions, the brain microvascular endothelium is joined by tight junctions consisting of claudins, occludin, or junctional adhesion molecules, with the support of adherens junctions, a specific basement membrane, perivascular astrocytic foot processes, and pericytes, forming the neurovascular unit (Huang et al. 2025). In turn, the neurovascular unit strictly controls paracellular or transcellular passage by employing specific solute carriers, receptors, or efflux transporters for ionic/molecular homeostasis or the exclusion of blood-borne proteins/toxins (Nájera-Maldonado et al. 2025). In the process of epileptogenesis with recurrent seizure activity, the inflammatory mediators, such as IL-1β or HMGB1, as well as the process of oxidation, lead to the activation of the cerebral microvascular endothelium with the upregulation of adhesion molecules, disruption of the tight junction, cytoskeleton rearrangements, or the process of transcytosis, thereby leading to the disruption of the BBB (Geng et al. 2025).

The disruption of the BBB leads the way for the crossing of the serum proteins such as albumin, prothrombin, or plasminogen into the brain parenchyma or the peripheral immune cells (Archie et al. 2021). The binding of albumin with the astrocytic receptors, such as transforming growth factor beta (TGF-β), leads to astrogliosis with downregulations of Kir.4.1 or glutamate transporter (Archie et al. 2021). In contrast, the cerebral microvascular endothelium releases the cytokines, chemokines, or VEGF due to seizure activity or hypoxic injury, thereby further exacerbating the neuroinflammation (Li et al. 2025b). VEGF signaling leads to the acute capillary/hepatic permeability/increased neurotrophic support or vascular leakage (Lee et al. 2025). VEGF signaling with the abnormalities in the structure leads to the development of the capillary system with a dissimilar structure (Lee et al. 2025). Microglia, in interaction with the astrocyte and the cerebral microvascular endothelium leads to the modulation of the newly developed capillary with blood or the modification of the blood flow with the cerebral activity (Sun and He 2025). Therefore, endothelial cells are not merely passive channels but active modulators that, through integrity mechanisms, inflammation, and vascular/metabolic coupling, may play a substantial role in influencing whether a transient insult progresses to a definitive epileptic pathologic condition.

### Cytokine and chemokine mediators of inflammation

**Fig. 2:**
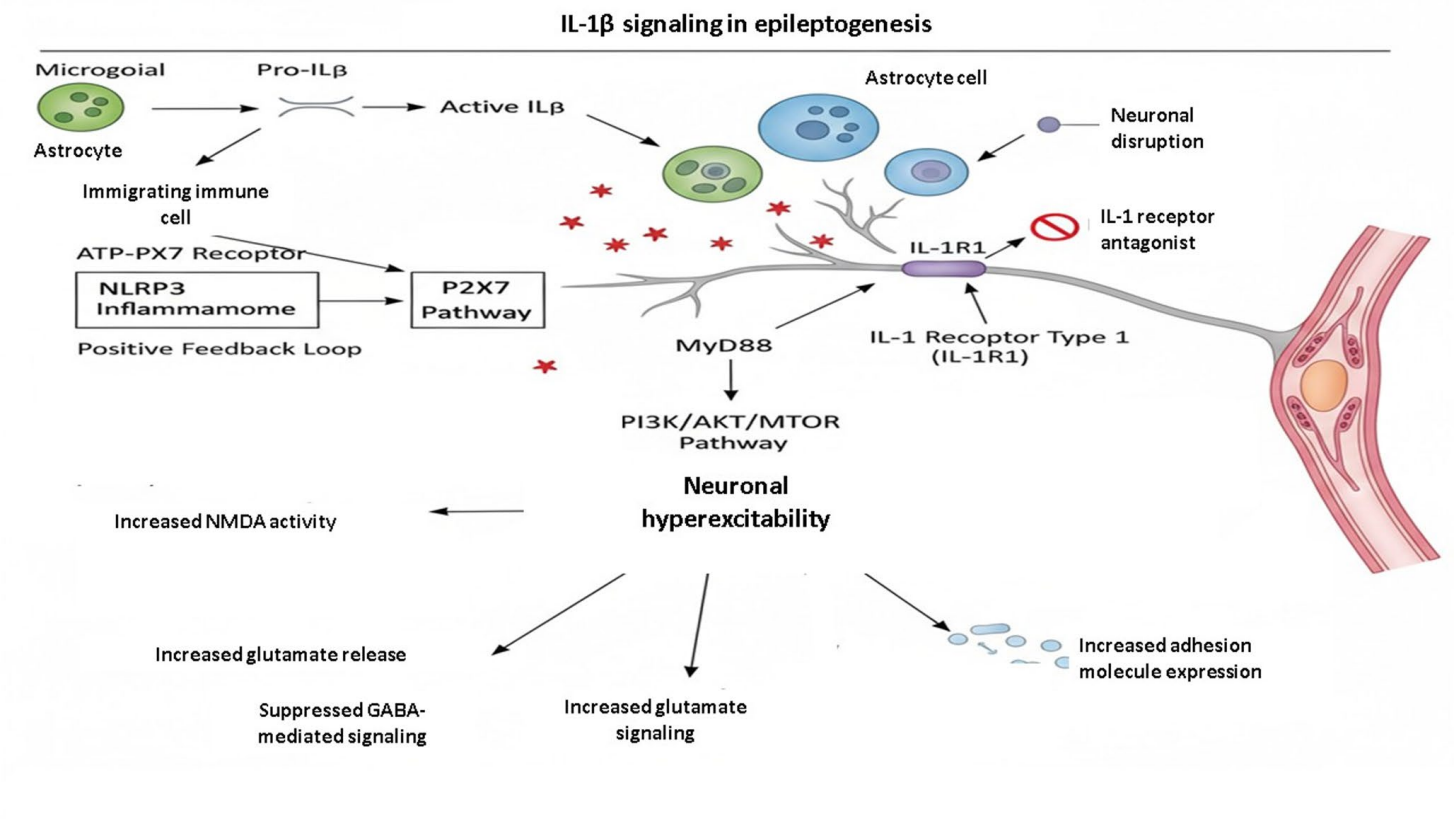
The role of il-1β signaling in epileptogenesis. This schematic highlights how interleukin-1β (IL-1β) acts as a central driver of neuronal hyperexcitability. The process begins when microglia, astrocytes, and immune cells produce pro-IL-1β, which is activated through the NLRP3 inflammasome and P2 × 7 receptor pathways. Once active, IL-1β binds to neuronal IL-1R1 receptors, triggering intracellular cascades, specifically MyD88 and PI3K/AKT/mTOR, that shift the brain’s chemical balance. By increasing glutamate release and NMDA receptor activity while simultaneously suppressing GABAergic inhibition, these pathways create a hyper-responsive neural environment conducive to epilepsy. Notably, the IL-1 receptor antagonist acts as a natural brake on this system by blocking IL-1R1 activation

Cytokines and chemokines play important roles as molecular messengers to convert seizures and brain injury into chronic neuroinflammatory pathways to drive epileptogenesis (Fig. 2) (Thergarajan et al. 2025). These factors are primarily expressed in microglia, astrocytes, neurons, and endothelial cells through distinct receptors to regulate synaptic function, BBB integrity, as well as gene expression networks to establish network excitability (Thergarajan et al. 2025). Among these factors, IL-1β, TNF-α, IL-6, TGF-β, as well as damage-associated molecular patterns including HMGB1, as well as chemokines CCL2 and CXCL10, have been most clearly linked to human epilepsy as well as animal models (Tastan and Heneka 2024; Shi et al. 2025).

IL-1β is among the pro-inflammatory cytokines that have been extensively investigated in the context of epilepsy and is upregulated in the brain, cerebrospinal fluid, and serum of patients suffering from drug-resistant epilepsy (Yu and Sun 2025). IL-1β is secreted mainly in its precursor form by microglia, astrocytes, and immigrating immune cells, which is then cleaved into the active form by the enzyme caspase-1 in the form of the NLRP3 inflammasome, thereby signaling via the IL-1 receptor type 1 (IL-1R1) receptor (Yu and Sun 2025). The binding of IL-1β to IL-1R1 triggers the recruitment of the adaptor molecule MyD88, thereby initiating the downstream signaling pathways involving the phosphoinositide 3-kinase (PI3K)/protein Kinase B (AKT)/mTOR and NF-κB pathways, ultimately resulting in the transcription of other pro-inflammatory genes, including the gene-encoding NLRP3, thus forming a positive feedback loop that maintains the inflammation within the neuronal microenvironment (Yu and Sun 2025). IL-1β has multiple downstream mechanisms that result in neuronal hyper-excitability, which occur through the increased activity of the NMDA receptor via the ceramide-mediated phosphorylation of the NR2B subunit, increased calcium influx, and release of glutamate, along with the suppression of GABA-mediated signaling (Geng et al. 2025). IL-1β also impairs the integrity of the BBB, thereby increasing the expression of adhesion molecules that favor the migration of leukocytes across the BBB (Gryka-Marton et al. 2025). IL-1β signaling can be blocked by the administration of IL-1 receptor antagonists or inflammasome inhibitors, thus providing concrete evidence of the role of IL-1β, a pro-inflammatory mediator, in disease modification of epileptogenesis, and not merely serving as a biomarker (Gryka-Marton et al. 2025).

This IL-1β-mediated hyperexcitability is of particular clinical relevance in catastrophic epilepsy syndromes such as FIRES and NORSE, where massive cytokine storms drive refractory status epilepticus. The successful clinical use of the IL-1 receptor antagonist Anakinra in these patients provides a ‘bedside-to-bench’ validation of the MyD88 pathway as a viable therapeutic checkpoint (Lai et al. 2020).

Another prominent cytokine is TNF-α, which is quickly increased in epileptic foci and blood/cerebrospinal fluid (CSF) following seizures, primarily produced in activated microglia and astrocytes, and also in stressed neurons (Chadwick et al. 2008; Foiadelli et al. 2023). The actions of TNF-α are mediated by the interaction with its receptors, TNFR1 and TNFR2, and activation of NF-κB and caspase-dependent pathways that have both neurotoxic and context-dependent neuroprotective roles (Chadwick et al. 2008). The role of the TNF-α in the epileptic brain is centered on the promotion of hyperexcitability, mainly as a post-synaptic receptor modulator. The mechanism entails the cell surface expression and facilitation of the phosphorylation of AMPA receptors, especially the GluA2-deficient calcium-permeable variants, and the reduced cell surface expression of GABA_A_ receptors (Chen et al. 2021). Additionally, the release of glutamate from astrocytes and the downregulation of glutamate transporters are increased (Cuellar-Santoyo et al. 2023). Furthermore, at the neurovascular junction, the presence of TNF-α mediates endothelial activation and the increased expression of adhesion molecules and the BBB leakage that dramatically potentiates the albumin leakage into the interstitial space and the activation of insoluble astrocytic TGF-β signaling (Fang et al. 2023). On the other hand, the actions of the activated TNFR2 mediate the promotion of neuronal plasticity and survival in certain contexts. Thus, generalized inhibition of the actions of TNF-α would have several consequences (Papazian et al. 2021).

**Fig. 3:**
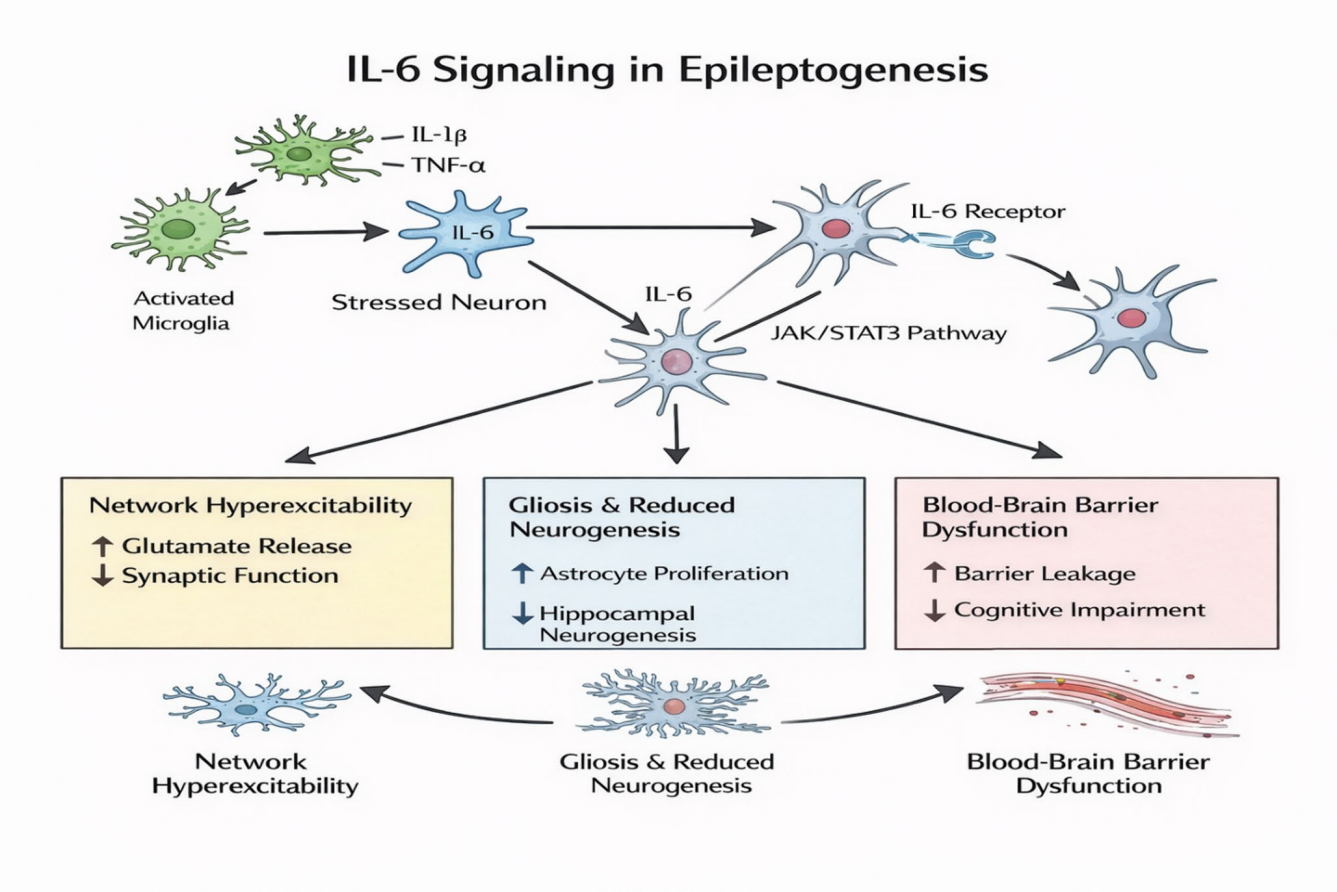
Il-6 signaling pathways in the pathogenesis of epileptogenesis. This diagram details how interleukin-6 (IL-6) acts as a pivotal mediator of the long-term neurological shifts seen in epilepsy. Triggered by stressors and cytokines like TNF-α and IL-1β, activated microglia and neurons secrete IL-6, which then engages the JAK/STAT3 signaling pathway. This activation precipitates a triple threat of pathological changes: it disrupts synapses by ramping up glutamate release, reshapes brain structure through gliosis and impaired neurogenesis, and compromises the blood-brain barrier via CCL2. Together, these mechanisms transform a localized inflammatory response into a broader state of network hyperexcitability and cognitive decline

IL-6 plays multiple roles as a cytokine, which can either stimulate or support nerve growth, but its major epileptogenic properties follow persistently elevated circulating blood levels of this molecule (Fig. 3) (Kummer et al. 2021). Under physiological conditions, there appears to be a normally low level of production of this protein, where, following seizures or injuries in the CNS, there appears to be a marked over-expression of this protein in astroglia, microglia, as well as in neurons, which can follow the production of several molecules, including IL-1β, TNF-α, as well as IL-17 (Kummer et al. 2021). IL-6 activates its target receptor, the IL-6 receptor (IL-6R) in combination with gp130, thereby activating the Janus kinase (JAK)/signal transducer and activator of transcription 3 (STAT3) pathway, as well as MAP kinases, which influence gene transcription that can regulate synaptic functions, gliosis, and neurogenesis (Mihara et al. 2012; Hu et al. 2021). It appears to be experimental evidence that this protein can upregulate glutamate release, downregulate long-term potentiation, reduce hippocampal neurogenesis, as well as induce gliosis, most of which can influence network hyper-excitability as well as cognitive manifestations (Mihara et al. 2012). Also, there appears to be an increased level of this protein in individuals with temporal lobe epilepsy, as well as coherence between this protein level and increased frequency of seizures, as well as failure of pharmacological therapy in various instances (Peltola et al. 2023). It has been demonstrated that interaction between this protein and chemokine production, where this protein can induce production of chemokines, as well as infiltration of leukocytes, most of which can contribute to an increased antiepileptogenic inflammatory milieu that can follow central nervous system seizures (Grebenciucova and VanHaerents 2023).

The translational significance of the IL-6/JAK-STAT3 axis is most evident in TLE, where persistently high levels of IL-6 in the serum and CSF correlate with increased seizure frequency and a high degree of pharmacoresistance. This suggests that IL-6 may serve as a biomarker for ‘inflammatory-metabolic phenotypes’ that are less likely to respond to traditional ion-channel-targeting ASMs (Chen et al. 2025).

HMGB1 is a cytokine-like pattern recognition molecule that has been increasingly identified as the key regulator of the inflammatory response in epilepsy (Geng et al. 2025). Although it is known to be a nuclear protein that maintains the organization of chromatin in the cell in the resting state, it is actively released from neurons, astrocytes, and microglia under the conditions of epilepsy, oxidative stress, and inflammation caused by the presence of IL-1β and/or TNF-α (Geng et al. 2025). Following the release into the extracellular space, HMGB1 forms a complex that binds to pattern recognition receptor molecules TLR-2 and, to a lesser extent, to TLR-4 and the receptor for advanced glycation end-products (RAGE) present on the surface of neurons and glia, as well as on the BBB endothelial cells (Li et al. 2025a). The HMGB1 signaling in the CNS leads to the activation of the transcription factor NF-κB and the MAPK signaling pathways; in turn, this leads to the upregulation of the production of the pro-inflammatory cytokines (IL-1β, IL-6, and TNF-α), as well as the upregulation of the NLRP3 inflammasome in the CNS, generating the feed-forward loop of the innate immune response (Mao et al. 2022; Yang et al. 2025). Specifically, in the neurons, the signaling by HMGB1 and the TLR-4 leads to the increased NMDA receptor function through the enhancement of NR2B receptor phosphorylation and the increased values of Ca2 + influx, and thereby, to the increased sensitivity to the generation of seizures and the values of epilepsy (Zhang et al. 2022). On the other hand, in the BBB endothelial cells, the signaling by HMGB1 leads to the breakdown of the BBB and the presence of the serum protein albumin in the CNS through the further activation of the TGF-β signaling pathway as the mechanism of its breakdown (You et al. 2023).

Chemokines represent another large category of inflammatory mediators in epilepsy, serving as chemoattractive cytokines involved in the regulation of leukocyte trafficking, in addition to their direct effects on neuronal and glial cells (Milano et al. 2024). Many chemokines, such as CCL1, CCL2, CCL4, CCL5, CCL22, CXCL10, and CXCL12, are upregulated in the epileptic brain, often in perivascular areas and glial cells (Tröscher et al. 2021). The role of the CCL2-CCR2 pathway in particular has been drawn attention to: CCL2 secreted from astrocytes, microglia, and endothelium chemoattracts CCR2 + monocytes and T cells from the periphery to the epileptic brain after SE, where it secretes other cytokines, contributes to neuronal damage, and favors the remodeling of epileptogenic networks (Tröscher et al. 2021). Experimental data indicate that the engagement of the CCL2-CCR2 pathway also leads to IL-1β secretion through STAT3 and inflammatory responses in neurons, linking chemokines to major cytokines (Tian et al. 2017). CXCL10, which is primarily secreted from astrocytes and microglia in response to interferons and IL-1β, binds to CXCR3 + T lymphocytes and microglia to promote their trafficking and microglial activation at epileptic foci (Petrisko et al. 2020). Some chemokines, such as CXCL12, also modulate neuronal migration, pathfinding, and synaptic plasticity, with their dysregulation following seizure activity contributing to dysregulated neurogenesis and mossy fiber sprouting (Song et al. 2023). Because chemokines and their receptors represent largely non-redundant families, it might be necessary to use these ligands or receptors individually or together with other anti-cytokines or BBB-directed therapies to modify disease (Xu et al. 2023). Chemokine represents another large category of inflammatory mediators in epilepsy, serving as chemoattractive cytokines involved in the regulation of leukocyte trafficking, in addition to their direct effects on neuronal and glial cells (Xu et al. 2023).

In sum, cytokine and chemokine mediators comprise an interrelated inflammatory cascade in epileptogenesis, where IL-1β, TNF-α, IL-6, TGF-β, HMGB1, and chemokines CCL2 and CXCL10 participate in self-reinforcing loops of pro-inflammatory bioactivations of neuroinflammation, BBB breakdown, and neuronal hyperexcitability in feed-forward loops. The temporal dynamics of their induction, acute and persistent following injury, and throughout the latent and chronic phases, make them mechanistic mediators and also signals for markers of the process of epileptogenic evolution and maturation (Reddy et al. 2025). Novel approaches using selective modulation of these pathways, including IL-1R antagonists, HMGB1 antagonists, anti-IL-6 treatments, TGF-β antagonists, and chemokine receptor antagonists, are vigorously pursued as possible disease-modifying interventions that might prevent the permanent establishment of the inflammatory cascade within the epileptic network (Reddy et al. 2025).

### Blood–brain barrier disruption and immune activation

The disruption in the BBB is now recognized as an active process rather than a passive result in epileptogenesis per se, rendering a highly controlled neurovascular niche into an obligatory pro-excitatory and pro-inflammatory drive (Han et al. 2024). Under physiological conditions, due to the highly specialized tight junctions in brain microvascular endothelia composed of pericytes and astrocyte foot processes, paracellular transport is limited to tight spaces in order to tightly regulate entry of ions, proteins, and immune elements across into the brain parenchyma (Han et al. 2024). Acute stimulation in shear damage from brain injury due to trauma, ischemia following stroke, inflammation due to infections, as well as epileptic activity, are known to increase oxidative damage to endothelia due to inflammation from infections, as well as damage from trauma to brain injury, in addition to epileptic activity (Han et al. 2024).

Experimental as well as clinical imaging using Contrast MRI as well as Evans Blue dye in various models following status epilepticus as well as brain injury clearly indicate an early increase in permeability following brain injury as well as during status epilepticus as well as an association between degree as well as regional area of damage in BBB permeability as an indicator for an increased risk of developing epilepsy in respective subjects (Kaur et al. 2011; Bar-Klein et al. 2017). The critical step following damage to BBB perturbs transport as well as infiltration into brain parenchyma from an albumin-fibrinogen rich circulation in particular in hippocampus as well as neocortex due to active uptake from astrocytes as well as perivascular elements following which albumin binds to transforming growth factors receptors thus initiating signaling through Smad as well as MAPK pathways to transcriptionally reprogram expression in astrocytes to selectively downregulate expression of Kir4.1 potassium as well as aquaporin 4 channels thus perturbing potassium as well as water homeostasis as well as thus facilitating neuronal discharge (Zapata-Acevedo et al. 2022; Suleymanova and Karan 2025).

Simultaneous activation in transforming growth factors signaling due to albumin in astrocytes in particular facilitates development of novel excitatory synapses due to increased density of cortical as well as hippocampal spine formation in addition to novel connectivity, as indicated in particular in various models where direct cortical administration of either albumin or transforming growth factors activates an increased incidence of spontaneous discharge in subjects (Zapata-Acevedo et al. 2022).

Immune activation, encompassing central innate immunity and peripheral adaptive responses, intersects with BBB dysfunction as an important driving force in the process of epileptogenesis (Shi et al. 2025). Micro- and astroglia within the central nervous system react to seizures and the consequent leakage of the BBB in micro/millisecond activated responses, resulting in the release of a multitude of cytokines (IL-1β, TNF-α, IL-6), chemokines (CCL2, CXCL10), and damage-associated molecular mediators like HMGB1, these in turn influence neuronal excitability and plasticity (Qin et al. 2023). These molecules (IL-1β and HMGB1), respectively interacting with IL-1R1 and TLR4/RAGE, sensitize and increase NMDA receptors and the release of glutamate, and the action of TNF-α increases the insertion of Ca2+-permeable AMPA and GABA_A_ receptors into the membrane and the internalization of GABA_A_ receptors (Zhang et al. 2022). On the other hand, chemokines like CCL2 and CX10 create a chemotactic gradient for peripheral immune monocytes and T cells to enter the CNS (Heng et al. 2022). These leukocytes enter the CNS from the blood and are activated as a consequence of peripheral inflammation. In temporal lobe epilepsy, effector T cells are found to be activated with increased expression of activation markers (CD69, CD25), and there are alterations in the regulatory T cell ratios in the peripheral blood (Heng et al. 2022). These peripheral leukocytes interact with micro/astroglia in the CNS and produce ROS and toxic mediators (Heng et al. 2022).

On the other hand, there are immune responses, as in certain experimental models, which are important in the abolition of seizures and in the healing of the CNS (Sanz et al. 2024). Activation can be an important driving force in the consequence of epilepsy as a consequence of the genetic predisposition for seizures and the consequent BBB leakage; in that case, the immune response produces a lowering in the seizure threshold and an important activation in the hyperexcitability of the CNS (Sanz et al. 2024). Hence, in the treatment of epilepsy, certain modulators have been developed that interact with the immune activation at various levels. These include agents for anti-IL-1R therapy, anti-HMGB1 inhibitors, anti-IL6 therapy, anti-TGF-β therapy, and anti-leukocyte trafficking and anti-co-stimulatory molecules.

## Crosstalk between metabolic stress and neuroinflammation

### Bioenergetic signaling pathways (AMPK, mTOR, NF‑κB)

The bioenergetic signaling pathways involving AMPK, mTOR, and NF-κB play a very important link between mitochondrial metabolism and neuronal excitability in the context of epileptogenesis (Xu et al. 2025). These pathways are involved in the detection of energy and redox status and inflammatory signals in cells and direct appropriate modification of protein synthesis and degradation and neuronal plasticity according to whether the insult heals or leads to chronic epilepsy (Xu et al. 2025).

AMPK is a cellular sensor of energy that is activated in response to an increase in the AMP/ATP, or ADP/ATP, ratio, such as that caused by seizure activity, hypoxia, or mitochondrial dysfunction, and serves to maintain energy balance through the inhibition of anabolic pathways and the promotion of catabolic pathways (Garcia and Shaw 2017). In neurons and glial cells, the activation of AMPK suppresses protein synthesis and cell growth, upregulates autophagy, increases glucose uptake and fatty acid oxidation, and promotes mitochondrial biogenesis through the activation of PGC-1α, to preserve energy homeostasis and protect against metabolic insults (Garza-Lombó et al. 2018). In models of status epilepticus, seizure-induced ATP depletion activates AMPK and upregulates the BH3-only protein Bmf, although interestingly, AMPK inhibition blocked Bmf upregulation but aggravated neuronal death, suggesting that activated AMPK contributes to protective, survival responses to seizure activity (Moran et al. 2013). By contrast, chronic or excessive AMPK activation contributes to neuronal apoptosis or maladaptive synaptic changes according to context, suggesting a complex role in epileptogenesis (Yang et al. 2022). AMPK downregulates mTORC1 through phosphorylation of TSC2 and Raptor proteins, thus playing an inhibitory role in mTOR-mediated protein synthesis and growth (Garza-Lombó et al. 2018). In infantile epileptogenesis, the appearance of a Warburg-like glucose metabolomic profile with aerobic glycolysis suppresses AMPK and derepresses mTOR activity, contributing to the emergence of a pro-epileptogenic environment characterized by excessive biosynthesis or network remodeling (Alqurashi et al. 2021). As for pharmacotherapy, the use of activators of AMPK, such as metformin, suppresses seizure activity and ameliorates seizure intensity across models, suggesting a role through mTOR pathway inhibition, reduction of oxidative injury, mediated through mTOR inhibition, reduced neuroinflammation mediated through inhibition of the NF-κB pathway, or preserved mitochondrial energetics or bioenergetic hemodynamics (Lin et al. 2023; Troise et al. 2024).

mTOR is a master regulator of growth, metabolism, and autophagy, integrating inputs from growth factor stimulation, amino acid availability, hypoxia, and energy status through two main protein complexes, mTOR complex 1 and mTOR complex2 (Panwar et al. 2023). While mTOR Complex1 is known to phosphorylate and stimulate protein synthesis and biosynthesis, and reduce autophagy, mTOR Complex2 is implicated in cytoskeletal organization and Akt signaling, and is also implicated in neuronal development, plasticity, and traumatic injury (Panwar et al. 2023). In epilepsy, excessive mTOR activation is recognized as one characteristic feature of mTORopathies, such as in tuberous sclerosis, focal cortical dysplasia, and some cases of hemimegaloencephaly, due to germline and/or somatic mutations in TSC1/2, PTEN, and similar genes that constitutively activate mTOR complex1 signaling and drive mTOR-mediated neurodevelopmental anomalies and epilepsy in early developmental stages and often involves intractable seizures (Moloney et al. 2021; Haile et al. 2025).

In addition, mTOR has also become activated in models of acquired epileptogenesis, such as kainate pilocarpine models and traumatic brain injury models of epilepsy, which is transient yet strong and generally peaks days after status epilepticus and is found coupled to a period of strong synaptic plasticity and gliosis (Zeng et al. 2009; Sumadewi et al. 2023). Meanwhile, excessive mTOR activation has resulted in dysregulated sprouting and growth in abnormal dendrites and synapse density toward enhanced expression in synaptic protein and channel biosynthesis and in suppression of autophagy flux, thereby promoting offending intracellular inclusions, which trigger seizure hyperexcitability and neurodegeneration (Sumadewi et al. 2023). Additionally, in mTOR-mediated epileptogenesis, Warburg-like metabolism induced in episodes of epileptogenesis is driven in key part by Wnt signaling and enhanced aerobic utilization and tricarboxylic metabolism anomalies and independently inhibits AMP kinase and mTOR activation, thus suggesting that metabolic structural perturbations initiating mTOR are colloquially termed the key initial stimulus in causing network reorganization (Alqurashi et al. 2021). Rapamycin and similar mTOR inhibitors such as Everolimus and Sirolimus have already shown profound protective effects upon epileptogenesis and seizure activity in various models in which seizures are decreased, and cortical lesions are reduced, though persistent mTOR inhibition has resulted in impaired physiological plasticity/metabolism and/or immune and similar up regulatory actions toward regular growth in purely acquired seizure disorders (Boff et al. 2025).

Pathological mTOR overactivation is the defining feature of mTORopathies, a group of malformations of cortical development that include Tuberous Sclerosis Complex (TSC), focal cortical dysplasia, and hemimegaloencephaly. In these syndromes, genetic mutations in TSC1/2 or PTEN lead to constitutive mTOR signaling, resulting in both structural brain abnormalities and intractable seizures that often necessitate mTOR inhibitors like Everolimus for management (Man et al. 2024).

NF-κB is an inducible family of transcription factors that plays a pivotal role in regulating inflammation and oxidative responses, as well as in modulating cell survival pathways, thus linking bioenergetic and redox changes with genetic expression programs during epileptogenesis (Cai and Lin 2022). In the resting state, NF-κB dimers, typically p65/p50 heterodimers, are retained in cytoplasmic sequestered compartments by inhibitory IκBs, whereas distinct stimuli, including inflammatory cytokines (TNF-α and IL-1β), ligands for pattern recognition receptors (HMGB1 and lipopolysaccharide (LPS), oxidative stress, and glutamatergic excitotoxicity activate the IKK kinase complex, leading to the phosphorylation and degradation of IκBs and NF-κB nuclear translocation (Cai and Lin 2022). Once activated, NF-κB binds specifically to κB sequences in the promoters or enhancers of numerous genes encoding cytokines (IL-1β, TNF-α, and IL-6), chemokines (CCL2 and CXCL10), adhesion molecules, inducible nitric oxide synthase, subunits for NADPH oxidase, and anti- and pro-apoptotic proteins, including Bcl-2 family members and regulators of synaptic plasticity and neurogenesis (Cao et al. 2024). In epilepsy, NF-κB is activated in neurons, astrocytes, and microglia of the epileptic focus following status epilepticus and contributes both to neuronal protection and injury, depending on the type of cells, temporal relationship, and intensity of NF-κB signals (Foiadelli et al. 2023).

NF-κB stimulates cytokine expression that elevates neuronal excitability and BBB permeability, establishing in turn an autoregulatory loop as NF-κB-stimulated cytokines further activate NF-κB via glial and neuronal receptors (Gryka-Marton et al. 2025). Infection and inflammation-induced oxidative stress, driven by mitochondrial dysfunction and NADPH oxidase enzymatic activity, also accelerate NF-κB activation, whereas NF-κB target genes, including NOX2 and iNOS, enhance ROS and RNOS concentrations, firmly placing NF-κB in the vicious cycle of oxidative stress and inflammation in epilepsy (Lingappan 2018). Simultaneously, NF-κB transcription also upregulates cytoprotective genes, including Mn-SOD and Bcl-2, expressing support for maintaining mitochondrial and neuronal functions, indicating that complete NF-κB inhibition might be counterproductive in this scheme (Sohur et al. 1999). From the point of view of bioenergetics, NF-κB modulates metabolism in active glial cells and immune cells accumulating in inflammation and stimulates glucose and lactate secretion, which in turn could maintain and enhance inflammation, leading to changes in the bioenergetic background of epilepsy tissue layers (Jembrek et al. 2021). Inhibition of NF-κB signals using IKK blockers, decoy oligodeoxynucleotides, and plant-derived compounds effectively lessens the occurrence of seizures, as well as the expression levels of inflammation mediators, and also reverses neuron loss in models of epilepsy, highlighting the targetability of NF-κB in the development of new treatments for epilepsy (Mahjoubin-Tehran et al. 2025).

Together, it is hypothesized that the involvement of AMPK, mTOR, and NF-κB creates a self-reinforcing loop where the downstream components mTOR and NF-κB, governed by the status of energy, regulate, through the use of AMPK, while NF-κB-driven inflammation and oxidative stress, which feed back on mitochondrial biochemistry and the mTOR pathway, contribute to a loss of fine control, which, by over-activation of mTOR, NF-κB-driven inflammation, or inadequate AMPK activation, could be a characteristic of epileptogenic tissues, providing several points of intervention.

### Critical appraisal of the evidence

Recent mechanistic insights linking metabolic signaling, neuroinflammation,and neuronal excitability in epilepsy have largely emerged from experimental models and therefore, require careful interpretation when extrapolated to human disease. Many of the pathways discussed in this review, including AMPK signaling, NF-κB activation, and mitochondrial metabolic remodeling, are supported primarily by preclinical studies using chemically induced or genetically engineered rodent models. While these models provide important mechanistic insights, they do not fully recapitulate the heterogeneity of human epilepsy syndromes, which vary widely in etiology, disease progression, and treatment response. For example, activation of AMPK has been shown in several experimental models to exert neuroprotective effects through enhancement of mitochondrial biogenesis and inhibition of excessive neuronal firing. However, sustained AMPK activation during chronic metabolic stress may also promote catabolic pathways that contribute to neuronal vulnerability, suggesting that its role may be context-dependent and temporally dynamic. Similarly, activation of the NF-κB signaling pathway is often described as a central driver of neuroinflammation in epilepsy, yet emerging evidence indicates that NF-κB signaling can also participate in adaptive immune responses and neuronal survival depending on the cellular context and duration of activation.

Another important consideration is the temporal dynamics of these pathways. Acute seizure activity may trigger transient inflammatory and metabolic responses that are initially protective, whereas persistent activation during epileptogenesis may promote maladaptive remodeling of neural circuits. Moreover, evidence from human studies remains relatively limited and often relies on indirect biomarkers, post-mortem analyses, or small clinical cohorts. Consequently, many proposed mechanistic links should be interpreted as associations rather than established causal relationships. Future translational studies integrating longitudinal clinical data, advanced neuroimaging, and molecular profiling will be essential to clarify the temporal sequence and causal relevance of these pathways in human epilepsy. Recognizing these limitations is critical for avoiding overly deterministic interpretations and for guiding the development of targeted therapeutic strategies (Table 3).

**Table 3:** Strengths and limitations of major molecular pathways implicated in epilepsy

Pathway	Evidence source	Key findings	Major limitations
AMPK signaling	Rodent seizure models, cellular studies, and limited human tissue analysis	Regulates neuronal energy metabolism, mitochondrial biogenesis, and neuronal excitability	Human clinical evidence is limited; most data are derived from kainate or pilocarpine rodent models
NF-κB signaling	Animal models, human epileptic brain tissue, and inflammatory biomarker studies	Central regulator of neuroinflammatory gene expression in epileptogenesis	Difficulty distinguishing causation from the inflammatory response to seizures
mTOR pathway	Genetic epilepsy models, pharmacologic inhibition studies, and clinical observations	Controls neuronal growth, synaptic plasticity, and epileptogenesis	Evidence is strongest in genetic epilepsies (e.g., tuberous sclerosis); less clear in acquired epilepsy
Mitochondrial dysfunction	Animal seizure models, patient mitochondrial biomarkers, and imaging studies	Increased oxidative stress, impaired ATP production, and altered neuronal metabolism	Biomarkers in humans are indirect; the causal relationship remains uncertain
Oxidative stress pathways (ROS/RNS)	Animal studies, biochemical assays in patients	Seizures increase reactive oxygen and nitrogen species, leading to neuronal injury.	Difficult to determine whether oxidative stress is a cause or a consequence of seizures
NLRP3 inflammasome activation	Rodent epilepsy models, limited human studies	Promotes neuroinflammation via IL-1β and IL-18 release	Translational evidence in humans remains sparse
Gut–brain axis/microbiome signaling	Animal microbiome manipulation studies, emerging human microbiome analyses	Microbial metabolites influence neuroinflammation, neurotransmitter balance, and seizure susceptibility	Human microbiome data are heterogeneous and influenced by diet, medication, and environment
Neurotransmitter imbalance (GABA–glutamate)	Extensive experimental and clinical evidence	Reduced inhibitory signaling and increased excitatory neurotransmission drive seizure activity	Mechanistic details vary significantly among epilepsy syndromes
Blood–brain barrier dysfunction	Animal models, neuroimaging studies, and human tissue samples	Increased BBB permeability allows inflammatory mediators to enter the brain tissue	The temporal sequence between BBB disruption and seizures remains unclear
Microglial activation	Animal models, histological analysis of human epileptic tissue	Microglia regulate inflammatory signaling and synaptic remodeling	Human evidence mostly observational

## Therapeutic strategies targeting the mitochondria‑inflammation axis

Interpretation of therapeutic strategies targeting metabolic and inflammatory pathways in epilepsy requires careful consideration of the level of clinical validation supporting each intervention (Idzikowska et al. 2026). While numerous compounds have been proposed as potential modulators of epileptogenic pathways, the strength of evidence varies substantially across different therapeutic categories. Some interventions, such as established antiseizure medications and mTOR inhibitors for specific genetic epilepsies, are supported by controlled clinical trials and regulatory approval, providing relatively strong translational evidence (Idzikowska et al. 2026). In contrast, several metabolic modulators and anti-inflammatory agents discussed in the literature represent repurposed therapeutic candidates that have shown promising effects in experimental models but remain supported only by limited clinical observations or small pilot studies (Idzikowska et al. 2026).

### Antioxidant and mitochondria‑protective therapies

Antioxidant and mitochondrial protective therapies aim to break the vicious circle of seizure-induced mitochondrial ROS overproduction and mitochondrial damage to DNA, respiratory chains, and membranes, thus incrementing seizure threshold and contributing to epileptogenesis (Ji et al. 2024). Evidence from genetically based models, such as SOD2-knockout mice that exhibit spontaneous seizure activity (Liang et al. 2012). Various antioxidant strategies have been tried; however, mitochondrial-targeted antioxidants appear to be the most effective strategy (Yamada et al. 2020). These include compounds such as MitoQ and SS-peptides that readily enter the mitochondrial matrix and directly reduce ROS at the site of synthesis, as well as classical antioxidants such as vitamins C and E, N-acetylcysteine, coenzyme Q10, and melatonin that can improve redox balance and support the endogenous function of mitochondrial antioxidant enzymes such as superoxide dismutase, glutathione peroxidase, and catalase (Yamada et al. 2020). Polyphenols such as resveratrol, curcuminoids, and lycopene with seizure-protective properties in animal models of epilepsy have been shown to reduce lipid damage and increase activities of respiratory complexes I, II, and IV, as well as to modulate glutathione (Łukawski and Czuczwar 2023). Nuclear factor erythroid 2-related factor 2 (Nrf2) inducers such as sulforaphane and synthetic triterpenoids (RTA-408) are another group of substances with a highly effective antioxidant gene program that enhances mitochondrial stability and ROS levels while suppressing seizure frequency and neuronal death in models of kainate and pentylenetetrazol-induced seizure models (Goodfellow et al. 2020). These antioxidants suppress ROS; however, they can also modulate mitochondrial permeability transition and stabilize cardiolipin content or promote mitophagy to prevent mt-DAMPs from causing seizure-induced neuroinflammation (Nahar and Sohag 2025).

Nrf2 functions as a master regulator of endogenous neuroprotection, coordinating transcriptional programs that govern glutathione synthesis, NADPH regeneration, mitochondrial resilience, and detoxification enzymes (Sandouka et al. 2023a). Emerging evidence from acute seizure models demonstrates that Nrf2 induction occurs predominantly in neurons rather than astrocytes, challenging the traditional view of glial-restricted antioxidant control (Sandouka et al. 2023a). Importantly, this neuron-enriched Nrf2 expression is sustained in chronic temporal lobe epilepsy, where it correlates with adaptive stress responses rather than purely reactive gliosis (Sandouka et al. 2023b). These findings position neuronal Nrf2 as an intrinsic counterweight to seizure-induced oxidative damage and mitochondrial dysfunction (Kumar Singh and Shekh-Ahmad 2024). From a translational perspective, pharmacological activation of Nrf2 represents a compelling disease-modifying strategy (Kumar Singh and Shekh-Ahmad 2024). Clinically approved agents such as dimethyl fumarate, already validated for long-term CNS use, enhance Nrf2 nuclear translocation and downstream cytoprotective gene expression, offering a realistic repurposing avenue to attenuate oxidative stress, neuroinflammation, and possibly epileptogenesis (Sandouka et al. 2023c).

Beyond canonical Nrf2 activation, complementary redox-modulating strategies may further strengthen the translational scope of redox-based disease modification (Singh et al. 2026). Thioredoxin-mimetic peptides and small-molecule thiol catalysts can directly restore redox buffering capacity, suppress protein S-nitrosylation, and limit mitochondrial ROS amplification independently of transcriptional reprogramming (Singh et al. 2026). In parallel, proteostasis-targeting approaches, such as enhancement of the unfolded protein response, autophagy induction, or selective proteasome modulation, address redox-driven protein misfolding and aggregate toxicity, which are poorly corrected by Nrf2 alone (Sheeni et al. 2026). Integrating these modalities highlights redox control as a systems-level therapeutic axis rather than a single-pathway intervention, broadening clinical applicability across neurodegenerative and epileptogenic disorders (Sheeni et al. 2026).

### Anti‑inflammatory and immunomodulatory approaches

Anti-inflammatory/immunomodulatory therapies aim at mitigating the cytokine/chemokine storm, connecting seizure activity with brain injury, neuroinflammation, BBB damage, and epileptogenic reorganization. Some of the most promising strategies involve the targeting of the IL-1β/IL-1R1 pathway due to the solid evidence that IL-1β increases NMDA receptor activity, releases glutamate, and increases seizure susceptibility, and is consistently found elevated in the epileptic brain tissues/CSF (Badawi et al. 2024b; Sanz et al. 2024). Anakinra, an IL-1 receptor antagonist, has already shown efficacy in a few pilot series and case reports of drug-resistant epilepsies, including Febrile-infection related epilepsy syndrome, reducing seizure burden or improving outcome in the setting of failure of conventional therapies (Lai et al. 2020; Yamanaka et al. 2021). Other IL-1 targeted biologics, such as canakinumab or rilonacept, have been utilized with success based on their past performance in the context of autoimmune diseases (Arnold et al. 2022). Current clinical evidence from pilot series shows Anakinra can reduce seizure burden by over 50% in refractory cases like FIRES, marking it as a leading candidate for targeted neuroinflammatory intervention (Yamanaka et al. 2021). The observation that IL-1 receptor antagonists can block these effects in animal models provides strong experimental proof that IL-1β is a modifiable contributor to the process, rather than a mere biomarker (Supino et al. 2022).

IL-6 and TNF-α also represent interesting targets, since IL-6 levels parallel seizure activity, resistance, or both, CI-3 and a critically excessive elevation of CI-3 has been found in refractory status epilepticus, for which CI-3 has been used off-label with partial success, whereas the efficacy of TNF inhibitors has been variable but promising in the case of autoimmune episodes (Madireddy and Madireddy 2023; Fang et al. 2024). More general immunomodulatory agents, including corticosteroids, intravenous immunoglobulins, plasmapheresis, or B-cell depleting agents (rituximab), have a recognized role in the case of autoimmune encephalitis with seizure activity, and can probably prevent the development of chronic epilepsy if started early (Madireddy and Madireddy 2023). Some antiseizure drugs, such as valproate and carbamazepine, have been shown with preclinical data, in a concentration-related fashion, capable of reducing the amount of main proinflammatory cytokines (IL-β, TNF-α, or IL-6) released by immune cells, providing an argument that antiseizure efficacy might have an immunomodulatory contribution (Andrzejczak 2011; Guo et al. 2025).

Other experimental anti-inflammatory strategies have involved COX2 inhibitors, statins, or minocycline, capable of interfering, respectively, with microglial activation, adhesion molecules, or matrix metalloproteins, with the dual aim of BBB repair or limiting the inflammatory cell traffic from the bloodstream into the CNS (Adibhatla and Hatcher 2010; Ahmadi et al. 2022). Tocilizumab has demonstrated partial success in refractory status epilepticus, where IL-6 levels are critically elevated, providing a clear path for late-preclinical to clinical transition (Jun et al. 2018). While preclinical models suggest that IL-6 can upregulate glutamate release and induce gliosis, the extent to which these mechanisms drive human epileptogenesis remains an area of active investigation (Li et al. 2023).

### Metabolic interventions: ketogenic diet, NAD⁺ boosters, and beyond

Such metabolic therapies include ketogenic dieting and so forth, aiming at the reprogramming of brain energy and redox homeostasis in a manner that promotes stable neuronal networks and inhibits hyperexcitability and inflammation (Sethi and Ford 2022). The ketogenic diet (KD), for example, which consists of a very high intake of fats, very low carbohydrates, and moderate protein, has long been employed for almost a century in the management of intractable epilepsy, and current cohorts indicate that 30–60% of patients enjoy ≥ 50% seizure reduction, and a proportion of these patients can also achieve seizure freedom (Sethi and Ford 2022). The mechanisms of the KD involve the inhibition of hyperexcitability and reduction of inflammation through the inhibition of neuronal ROS production, but the central hypothesis of the KD’s mechanisms involves the suppression of neuronal hyperexcitability and reduction of neuronal inflammation through the increase in mitochondrial biogenesis and respiratory capacity, reduction of ROS production, and the modulation of ATP-sensitive potassium channels, adenosine, and GABA production (Jang et al. 2024).

In addition to modulating ion and water transport, and thereby inhibiting hyperexcitability and neuronal inflammation, a more recent hypothesis involves the increase in nicotinamide adenine dinucleotide (NAD⁺) levels and the enhanced ratio of NAD⁺/NADH, thereby enhancing the activity of sirtuin and PARP signaling pathways, and the suppression of glycolysis in favor of oxidative phosphorylation (Jang et al. 2024). This, in turn, has led to the exploration of the direct modulation of the enhanced levels of nicotinamide adenine dinucleotide (NAD⁺) by using precursors such as nicotinamide riboside, nicotinamide mononucleotide, and niacin, all of which exert seizure-protective roles in epilepsy by enhancing the levels of brain NAD⁺, improving mitochondrial resilience, and a cocktail of drugs called the mitochondrial cocktail (Braidy et al. 2019) (Table 4).

**Table 4:** Clinical translation status of therapeutic strategies targeting metabolic and neuroinflammatory pathways in epilepsy

Therapeutic strategy	Example compounds	Mechanism of action	Evidence type	Level of clinical validation	Key limitations
Approved antiseizure medications	Valproate, levetiracetam, lamotrigine	Modulate neuronal excitability and neurotransmission	Large clinical trials and long-term clinical use	Approved therapy	Limited disease-modifying effects
mTOR inhibitors	Everolimus	Inhibits mTOR signaling involved in epileptogenesis	Clinical trials in tuberous sclerosis	FDA-approved for a specific epilepsy subtype	Not broadly effective across all epilepsy syndromes
Metabolic modulators	Metformin, resveratrol	Activation of AMPK and improvement of mitochondrial metabolism	Preclinical models, small clinical studies	Repurposed therapeutic candidates	Limited randomized clinical trial data
Anti-inflammatory agents	Anakinra, tocilizumab	Target cytokine-mediated neuroinflammation	Case reports and small cohort studies	Early clinical evidence	Small sample size and heterogeneous populations
NLRP3 inflammasome inhibitors	MCC950	Inhibits inflammasome-mediated inflammation	Animal models	Preclinical stage	Lack of human studies
Antioxidant therapies	N-acetylcysteine, coenzyme Q10	Reduce oxidative stress and mitochondrial dysfunction	Experimental models and small clinical studies	Limited clinical evidence	Mixed efficacy results

## Emerging disease‑modifying therapies

### Multi‑target drug development

The concept of multi-target drug development in epilepsy is born out of understanding that epileptogenesis is driven by an intricately interconnected set of mechanisms, including ion channelopathy, synaptic plasticity, mitochondrial stress, and neuro-inflammation, such that modulation of one node alone often leads to incomplete and transient outcomes (Xu et al. 2024). Many available antiseizure medications already target multiple sites, such as voltage-gated sodium channels, calcium channels, and GABAergic systems, while new generations of so-called designed multiple ligands aim to rationally integrate targets for excitability, metabolism, and inflammatory pathways in one single entity (Xu et al. 2024). Examples include cenobamate, which acts both as an enhancer of GABA_α_ receptor function and as an inhibitor of persistent sodium current (Sankar et al. 2026), and padsevonil, which targets multiple synaptic vesicle protein 2 (SV2) isoforms and GABA_α_ receptors, although this one has so far had more restricted clinical development (Muglia et al. 2020). Yet apart from targets in and around the synapse, future multi-target treatments aim to integrate anti-inflammatory strategies such as IL-1β and/or HMGB1 signaling inhibition, NF-κB activation inhibition, and modulation of microglial phenotype with direct modulation of mitochondrial function and oxidative stress (Mao et al. 2022; Geng et al. 2025; Shokr 2025a).

For example, experimental compounds such as VX-765, a caspase-1 inhibitor (Dey et al. 2016), and TAK-242, which targets TLR4 (Dong et al. 2022), demonstrate that modulation of upstream inflammatory pathway points could reduce acute seizures as well as chronic epileptogenesis, mainly when combined with traditional ASMs, suggesting that so-called network therapeutics may indeed be more effective in patients with DR epilepsy. Another promising area would be multi-target compounds that modulate simultaneously metabolic pathway sensors such as AMPK and mTOR while decreasing glial inflammation, to directly target and modulate metabolism and inflammation crosstalk simultaneously, as reviewed in recent overviews (Garza-Lombó et al. 2018). Ideally, multi-target drug development would target not merely seizure regulation and control, but would also attempt to reduce and directly modulate epileptogenic network reorganizations and comorbidities, and would necessarily be tightly coupled with biomarker-driven patient stratification to select and target distinct molecular phenotypes such as elevated HMGB1 and/or TLR4 expression and activity or strong metabolic dysfunctions with specific multi-modal treatments.

The integration of VX-765 (Caspase-1 inhibitor) with traditional ASMs represents the most advanced preclinical strategy for combined seizure control and disease modification, moving beyond single-node intervention toward comprehensive network stabilization (Maroso et al. 2011) (Table 5).

**Table 5:** A table demonstrates which targets are ready for the clinic versus those that are still purely experimental

Intervention category	Target/pathway	Current status	Translational impact
Intervention category	Ketogenic diet	Standard of care	High: 30–60% achieve ≥ 50% seizure reduction.
Intervention category	IL-1R (Anakinra)	Clinical pilots	High; effective in neuroinflammatory syndromes (FIRES).
Intervention category	Caspase-1 (VX-765)	Phase II clinicals	Moderate; proof-of-concept for upstream inflammation.
Intervention category	MitoQ / SS-peptides	Late preclinical	Emerging focuses on mitochondrial ROS at the source.
Intervention category	SV2A / GABA (Padsevonil)	Clinical development	Limited; restricted development due to clinical outcomes.

### Novel biomarkers for precision medicine

​New biomarkers in the realm of precision medicine in epilepsy aim to integrate the complex interactions between genetics, inflammation, metabolism, and network connectivity in order to provide more precise diagnosis, prediction of epileptogenesis, therapy choice, and treatment follow-up (Labate et al. 2026). Concerning the inflammation axis, blood and cerebrospinal fluid cytokines, danger signals, such as IL-1β, TNF-α, IL-6, HMGB1, TLR ligands, and interferon gamma (IFN-γ), are being investigated as biomarkers of seizures, resistance, and network plasticity (Wang et al. 2025). High levels of HMGB1, for example, were associated with medically intractable epilepsy, predicting response to therapies targeting either HMGB1 or TLR4 pathways (Soytürk et al. 2024). Metabolic biomarkers would then comprise lactate, particular acylcarnitines, amino acid patterns, or more sophisticated metabolite fingerprints derived from mitochondrial biopath, perturbations in carbohydrate metabolism, as well as impaired neurotransmitter recycling; in mitochondrial epilepsies, these would allow diagnosis as well as prediction of response to metabolic or gene therapies (McCann et al. 2021).

Neurogenetic biomarkers would range from point mutations in defined genes, such as SCN1A, DEPDC5, TSC1/2, or more, via polygenic risk scores, being more broadly applied for defining specific epilepsy syndromes, anticipating secondary complications, as well as selecting patients for therapies targeting particular pathways, such as mTOR inhibitors, or future gene therapies (Veerabathiran and Iyshwarya 2025). Additional biomarkers in the realm of imaging would further expand the boundaries of precision medicine applied in epilepsy. Precise imaging techniques, already being applied in advanced MRI, PET, or PET-related methodologies, would visualize BBB leakage, gliosis, particular examples of which would comprise TSPO-PET imaging, as would map network alterations of metabolism (Katiyar and Manish 2025).

## Challenges and future directions

The translational gap and the nature of clinical trials are significant barriers to the development of disease-modifying therapies for the treatment of epilepsy, based on the crosstalk between neuroinflammation and metabolism that has been elucidated. Most of the available information derives from animal models that rely on potent chemoconvulsants or focal lesions, which are only a rough approximation of the complexity of human epilepsy. In preclinical testing, the onset of treatment is before or shortly after the epileptogenic insult, whereas in the clinic, the diagnosis of epilepsy is made after the onset of spontaneous seizures, making the conduct of true prevention studies rather impractical from the viewpoint of logistics and ethics.

Most of the experimental therapeutic approaches, such as antioxidants, anti-inflammatories, or metabolic modulators, are administered in young, otherwise healthy animals, in controlled conditions, whereas, in the patient, the complexity of therapy, systemic diseases, and genetic factors, which affect the metabolism of the agent, are present. Finally, the outcome parameters are different for the two categories of research, whereas the former use the frequency of seizures for a period of weeks or structural abnormalities, which are primary, whereas the latter are obliged to ascertain the outcome of the therapy in terms of the burden of seizures, the more accurate definition of quality of life, cognitive function, and the absence of adverse effects during the period of years rather than the shorter period of weeks or a month. Finally, the structures of the authorities regulate a greater affinity for symptomatic antiseizure therapy rather than the expensive prevention therapy in the high-risk population, such as post-traumatic injury or post-ischemic injury.

Moreover, the problem of the preponderance of the modest or non-conclusive efficacy of antiepileptogenic therapy in the unselected population illustrates the significant gap between the present knowledge of the diseases and the real gap between the results of the experiments and the efficacy of the therapy, which is indicative of practical barriers, such as the heterogeneity of the population, the absence of biomarkers, such as inflammation or metabolic mediators, in addition to the significant biological complexity of the problem.

New research tools are now being used to fill this gap by enabling more sensitive and biologically relevant interrogation of crosstalk between neuroinflammation and metabolism. Using human induced pluripotent stem cell (iPSC)-derived neurons, astrocytes, microglia, and brain organoids harboring patient-specific mutations allows investigation into how mitochondrial dysfunction, oxidative damage, and inflammation interact to regulate network excitability in genetically defined systems. Novel imaging approaches, two-photon microscopy, multimodal MRI-PET imaging, allow in vivo visualization of microglia activation, astrocyte activation, blood-brain barrier disruption, and metabolism within epileptogenic networks in some cases simultaneously with EEG. Genetic actuators expressing calcium activity, ATP levels, oxidative status, as well as signaling pathways (AMPK, mTOR, NF-κB), provide an in vivo readout into how bioenergetics stress and inflammatory activation dynamics change during or following seizures on cellular to subcellular levels.

Single-cell to spatial transcriptomic analysis enables cell type-specific interrogation of cellular activity following seizures to delineate subsets of microglia, astrocytes, endothelia actively promoting or suppressing epileptogenesis while showing in which tissues genes encoding mitochondria function, pro-inflammatory, chemokine activity, or metabolites are coregulated in space-time. More representative models in animals employing chronic epileptogenesis models with symptoms of concomitant human-like depression or cognitive dysfunction, in elderly model animals, in animals employing systemic inflammation or metabolic dysfunction, are more representative of human conditions. Using systems bioanalytical models to consolidate this orthogonal set of research findings identifies overlapping nodes in these pathways in which inflammasomes, MtDAMP pathways, and metabolic nodes function as maximal convergence sites to act in multimodal treatments.

While the proposed trinity paradigm of targeting AMPK, mTOR, and NF-κB offers a conceptually robust framework for disease modification, its clinical implementation faces significant hurdles regarding feasibility, safety, and therapeutic precision. The biochemical crosstalk between these master regulators is inherently non-linear, meaning that simultaneous modulation of three major metabolic and immune sensors increases the risk of unpredictable drug-drug interactions and off-target effects. From a safety perspective, chronic mTOR inhibition is associated with serious systemic concerns, including immunosuppression and metabolic disturbances such as hyperglycemia, while prolonged NF-κB inhibition may prove counterproductive by suppressing vital cytoprotective genes like Mn-SOD and Bcl-2 that are essential for neuronal survival under stress. Furthermore, while AMPK activation appears promising, excessive or chronic manipulation of brain energy sensors can lead to maladaptive synaptic changes or unintended apoptosis. Because these pathways are fundamental to normal cellular physiology, they likely possess a narrow therapeutic window, where the dosage required to interrupt epileptogenesis may overlap with levels that impair healthy brain function or systemic immunity, presenting a far more complex pharmacological challenge than traditional anti-seizure medications.

The prospects for personalized and preventive therapies in epilepsy increasingly hinge on the integration of such knowledge and biomarkers. Personalized therapies might seek to stratify patients based on inflammatory-metabolic phenotype, that is, patients who exhibit high levels of circulating HMGB1, IL-1β, and TNF-α, BBB breakdown, and mitochondrial decay might be targeted preferentially to trials of IL-1R antagonists, HMGB1 or TLR4 antagonists, or mitochondria-directed antioxidants and metabolic therapies. Patients could be stratified using genetic analyses to identify monogenic epilepsies that could be targeted to definitive mTOR inhibition, gene therapy, and targeted metabolic diets, including the ketogenic and modified Atkins diets in mitochondrial epilepsies. Patients identified in high-risk populations, including those who suffered severe traumatic brain injury, intracerebral hemorrhage, and CNS infections, might be identified early in the course of BBB breakdown, inflammatory exaggeration, and metabolic derangement to allow temporally restricted preventive therapies (anti-inflammatory, antioxidants, or metabolic) before the eruption of spontaneous epilepsy.

The use of digital health technologies (wearables, home EEG, and seizure triggers to sample blood or cerebrospinal fluid) should improve the ability to follow biomarker values in real time and to titrate or adjust therapies in response to those values. Ultimately, multi-omic patterns analyzed via machine learning may help to identify unique endotypes of epilepsy that might respond to individually tailored strategies that combine anti-seizure, anti-inflammatory, mitochondrial, and metabolic therapies to change the course of disease rather than merely to control its symptoms. All this will be made possible in the setting of large longitudinal studies, standardized biomarker panels, and designed and executed adaptive clinical trials involving the best collaborative effort between basic scientists, clinical researchers, and data scientists, but may hold out the realistic promise of both personalized and genuinely preventive epilepsy therapies.

Another important consideration when interpreting mechanistic pathways in epilepsy is the temporal dynamics of molecular signaling, as biological responses during acute seizure activity may differ substantially from those observed during chronic epileptogenesis (Xu et al. 2025). Acute seizures trigger rapid metabolic stress, transient oxidative imbalance, and short-term activation of inflammatory pathways such as NF-κB signaling and microglial activation. In this early phase, several molecular responses may initially serve adaptive or protective functions, including restoration of metabolic homeostasis, clearance of cellular debris, and stabilization of neuronal networks. For example, transient activation of AMPK during acute metabolic stress can enhance mitochondrial function and promote cellular energy balance (Xu et al. 2025). However, when these signaling pathways remain persistently activated during recurrent seizures or chronic epilepsy, they may contribute to maladaptive processes such as sustained neuroinflammation, mitochondrial dysfunction, synaptic remodeling, and neuronal hyperexcitability (Xu et al. 2025). Similarly, inflammatory mediators that initially participate in protective immune responses may become drivers of chronic neurodegenerative and epileptogenic mechanisms when activation becomes prolonged (Xu et al. 2025). These observations highlight the importance of considering time-dependent effects when evaluating mechanistic evidence and therapeutic targets in epilepsy. Consequently, interventions targeting metabolic or inflammatory pathways may produce different outcomes depending on the stage of disease progression, emphasizing the need for longitudinal studies and stage-specific therapeutic strategies in epilepsy research.

## Conclusion

The mechanisms of chronic epileptogenesis are increasingly unraveled through the framework of mito-inflammatory convergence. Mitochondrial damage due to oxidative phosphorylation dysfunction, overproduction of ROS, and disturbances in ion homeostasis initiates innate immune responses, which in turn include activation of inflammasomes as well as disruption of the BBB. The central bioenergetics centers, namely, AMPK, mTOR, and NF-κB, orchestrate these processes to turn cellular pathways from a restorative to a maladaptive/seizure-susceptible direction. This comprehensive model of epileptogenesis validates why conventional anti-epileptic therapies, which mainly target symptomatic ion channels, lack any disease-modifying properties. The landscape is therefore swiftly shifting toward triple target or simultaneous therapies aimed simultaneously at mitochondrial dysfunction, redox disturbances, and inflammatory pathways. This novel “mito-pharmacotherapy” pipeline encompasses research on mitochondrially targeted antioxidants, NAD+-based metabolism therapies, ketogenic diets, as well as novel multi-target pharmacological molecules. Re-designing management strategies through this “trinity” paradigm will hopefully help to shift from mere symptomatic treatment to true prevention as well as disease modification in epilepsy; therefore, tremendous international funding is being mobilized into these new paradigms of comprehensive bioenergetics approaches to this major debilitating CNS disorder.

## Data Availability

All data used for the review article have been cited in the text.

## Abbreviations

A1 astrocytes: Neurotoxic A1‑like reactive astrocytes
A2 astrocytes: Neuroprotective A2‑like reactive astrocytes
AAV: Adeno‑associated virus
AEDs: Antiepileptic drugs
AICAR: 5‑Aminoimidazole‑4‑carboxamide ribonucleotide
AKT: Protein kinase B
AMPA receptor: α‑Amino‑3‑hydroxy‑5‑methyl‑4‑isoxazolepropionic acid receptor
AMPK: AMP‑activated protein kinase
ASMs: Anti‑seizure medications
ATP: Adenosine triphosphate
BBB: Blood–brain barrier
Bcl‑2: B‑cell lymphoma 2
cGAS‑STING: Cyclic GMP‑AMP synthase–stimulator of the interferon genes pathway
CCL1: C‑C motif chemokine ligand 1
CCL2 (MCP‑1): C‑C motif chemokine ligand 2 (monocyte chemoattractant protein‑1)
CCL4: C‑C motif chemokine ligand 4
CCL5 (RANTES): C‑C motif chemokine ligand 5 (regulated upon activation, normal T cell expressed and secreted)
CCL22: C‑C motif chemokine ligand 22
CCR2: C‑C chemokine receptor type 2
CNS: Central nervous system
COX‑2: Cyclooxygenase‑2
CSF: Cerebrospinal fluid
CXCL10 (IP‑10): C‑X‑C motif chemokine ligand 10 (interferon‑gamma‑induced protein 10)
CXCL12: C‑X‑C motif chemokine ligand 12
CXCR3: C‑X‑C chemokine receptor type 3
DAMPs: Damage‑associated molecular patterns
DEPDC5: DEP domain–containing protein 5 gene
DdCBE: DddA‑derived cytosine base editor
DRP1: Dynamin‑related protein 1
EEG: Electroencephalography
FIRES: Febrile infection‑related epilepsy syndrome
FIS1: Fission 1 protein
GABA: Gamma‑aminobutyric acid
GABA_B receptor: Gamma‑aminobutyric acid type B receptor
GFAP: Glial fibrillary acidic protein
GLAST: Glutamate–aspartate transporter
GLT‑1: Glutamate transporter‑1
GluA2: Glutamate ionotropic receptor AMPA type subunit 2
gp130: Glycoprotein 130
HMGB1: High mobility group box 1
iNOS: Inducible nitric oxide synthase
IL‑1β: Interleukin‑1 beta
IL‑1R1: Interleukin‑1 receptor type 1
IL‑6: Interleukin‑6
IL‑6R: Interleukin‑6 receptor
IFN‑γ: Interferon gamma
IP‑10: Interferon‑gamma‑induced protein 10 (see CXCL10)
iPSC: Induced pluripotent stem cell
JAK: Janus kinase
KD: Ketogenic diet
Kir4.1: Inward rectifier potassium channel 4.1
LPS: Lipopolysaccharide
MAPK: Mitogen‑activated protein kinase
MCP‑1: Monocyte chemoattractant protein‑1 (see CCL2)
MFN1/2: Mitofusin 1/2
Mn‑SOD: Manganese superoxide dismutase
MRI: Magnetic resonance imaging
mt‑DAMPs / mtDAMPs: Mitochondria‑derived damage‑associated molecular patterns
mtDNA: Mitochondrial DNA
mTOR: Mechanistic (mammalian) target of rapamycin
MyD88: Myeloid differentiation primary response 88
NLRP3: NOD‑, LRR‑ and pyrin domain‑containing protein 3 (inflammasome)
NMDAR /: N‑methyl‑D‑aspartate receptor
NO / RNOS: Nitric oxide / reactive nitrogen oxide species
NORSE: New‑onset refractory status epilepticus
Nrf2: Nuclear factor erythroid 2–related factor 2
OPA1: Optic atrophy 1
PARP: Poly (ADP‑ribose) polymerase
PET: Positron emission tomography
PGC‑1α: Peroxisome proliferator‑activated receptor gamma coactivator 1‑alpha
PI3K: Phosphoinositide 3‑kinase
PTEN: Phosphatase and tensin homolog
RAGE: Receptor for advanced glycation end products
RANTES: Regulated upon activation, normal T cell expressed and secreted (see CCL5)
ROS: Reactive oxygen species
SCN1A: Sodium voltage‑gated channel alpha subunit 1 gene
SE: Status epilepticus
STAT3: Signal transducer and activator of transcription 3
SUDEP: Sudden unexpected death in epilepsy
SV2: Synaptic vesicle protein 2
TGF‑β / TGF‑β1: Transforming growth factor beta/beta 1
TFAM: Mitochondrial transcription factor A
TLR: Toll‑like receptor
TLR2: Toll‑like receptor 2
TLR4: Toll‑like receptor 4
TLR9: Toll‑like receptor 9
TNF‑α: Tumor necrosis factor alpha
TNFR1: Tumor necrosis factor receptor 1
TNFR2: Tumor necrosis factor receptor 2
TSC1/2: Tuberous sclerosis complex 1/2
TS: Tuberous sclerosis
TSPO: Translocator protein (18 kDa)
VEGF: Vascular endothelial growth factor
